# Quantum topology emerges at near field

**DOI:** 10.1038/s41377-026-02319-0

**Published:** 2026-05-08

**Authors:** Zhenyu Guo, Yijie Shen

**Affiliations:** 1 Centre for Disruptive Photonic Technologies, School of Physical and Mathematical Sciences, Nanyang Technological University, Singapore, Singapore; 2 School of Electrical and Electronic Engineering, Nanyang Technological University, Singapore, Singapore

**Keywords:** Nanophotonics and plasmonics, Nanophotonics and plasmonics

## Abstract

Mapping the total angular momentum of light bound to nanophotonic structures enables the creation of single-photon states with rich topological textures. This approach opens new opportunities for generating high-dimensional entanglement and provides a promising route toward robust quantum information processing.

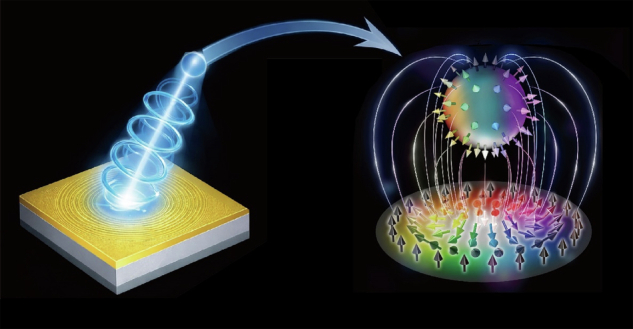

## Abstract (web summary)

Near-field nanophotonics platform with spin-orbital coupling enables single-photon skyrmions with robust topological structures for high-dimensional quantum information processing.

The quest for scalable quantum information processing has long been a tug-of-war between the demand for high-dimensional encoding and the intrinsic fragility of quantum states. Photons are excellent carriers of information, yet conventional encoding strategies—such as exploiting the polarization (spin angular momentum, SAM) or the spatial structure (orbital angular momentum, OAM) of structured light—often rely on bulky optical setups and remain susceptible to environmental noise^1–5^. Crucially, when light is confined to nanophotonic near-field environments, the traditional distinction between a photon’s SAM and OAM begins to break down. Instead, the two become intrinsically coupled, giving rise to a more fundamental quantity: the photon’s total angular momentum (TAM). Writing in ***eLight***, Amit Kam and colleagues report a significant advance: the realization of quasiparticle topological structures known as skyrmions in single-photon states mediated by nanophotonics^6^. Their work shows how the TAM of a single photon can be harnessed to weave complex, topologically structured patterns that may exhibit enhanced robustness.

The concept of skyrmions, originally introduced by Tony Skyrme in nuclear physics to describe stable, knot-like field configurations, has since spread across many branches of physics, including magnetism and condensed-matter physics. More recently, skyrmions were also discovered in optics and photonics^7,8^, and followed up by a series of papers^9–12^. In the quantum regime, optical skyrmions can generally be divided into two categories. One is local skyrmions, where the topology arises from the entanglement between different internal degrees of freedom of a single photon. The other is nonlocal skyrmions, whose topology is constructed from entanglement shared between two spatially separated photons^13–15^. Single-photon skyrmions have recently been realized using quantum emitters, including quantum dots^16^, nitrogen-vacancy centers^17^, and microrings^18^. Yet it would be highly desirable to be able to create single photon skyrmions in a nanophotonic chip, where the skyrmionic structure could be shaped and controlled by the near-field structure (Fig. 1).

**Fig. 1:**
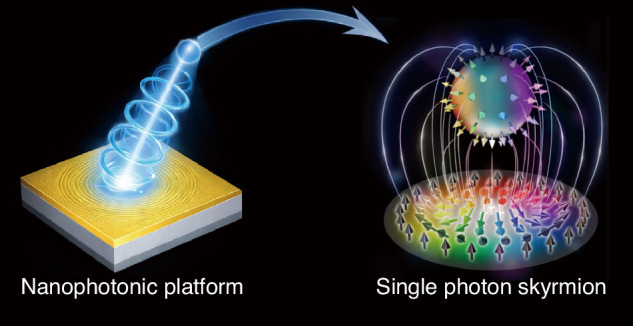
Conceptual illustration of the generation of a single-photon skyrmion mediated by a nanophotonic platform. A single photon interacts with a nanostructured surface, where near-field nanophotonic interactions couple its spin and orbital degrees of freedom into total angular momentum modes. After scattering back into free space, the photon emerges as a topologically structured polarization field forming a Stokes skyrmion

Recently, Kam et al. have addressed this challenge and demonstrated the generation of single-photon skyrmionic structures by shaping the near-field TAM of single photons at nanophotonic interfaces. In their work, the researchers employed a plasmonic platform consisting of a gold film patterned with nanoscale features that couple light into surface plasmon polaritons. At these subwavelength scales, the conventional distinction between SAM and OAM no longer holds. Instead, the near-field interaction binds them together into TAM modes. When a single photon interacts with the nanostructure, its state becomes confined to a specific TAM channel^19^. As the light is scattered back into free space, this state evolves into a high-dimensional entangled structure linking the photon polarization and spatial mode. Using quantum state tomography, the team verified that the resulting states exhibit a nontrivial topological texture in the distribution of the photon properties across the wavefront. The implications of this work extend beyond the demonstration of a new photonic structure and can be summarized in two key aspects.

1. A new physical picture of photon angular momentum in nanophotonic near-field environments. Under strong spatial confinement, SAM and OAM no longer behave as independent variables. Instead, they are intrinsically coupled through near-field interactions and are more naturally described by TAM modes that characterize the photon state.
2. A pathway for engineering complex quantum optical states on integrated photonic platforms. By exploiting surface plasmon polaritons in nanostructures, the angular momentum structure of photons can be manipulated directly in chip-scale devices. This capability provides a promising route toward generating and controlling high-dimensional quantum states in integrated quantum photonic technologies.

Although the experiment demonstrates remarkable control over nanophotonic light–matter interactions, it also leaves several intriguing directions for future exploration. For instance, it remains an open question whether similar mechanisms can be extended to generate skyrmion-like entanglement involving multiple photons, or whether such states could be exploited to simulate exotic quantum phases that are otherwise difficult to realize in laboratory systems. It also inspires the question of being able to implement on-chip switching between different states of quantum skyrmions. By enabling the generation of complex quantum photonic states in nanophotonic platforms, this work moves quantum topology beyond a purely theoretical concept and into the practical toolkit of quantum engineering. In particular, the ability to engineer structured quantum states in integrated nanophotonic devices may provide a promising route toward robust, high-dimensional quantum information processing and future large-scale quantum networks.
